# StructRNAfinder: an automated pipeline and web server for RNA families prediction

**DOI:** 10.1186/s12859-018-2052-2

**Published:** 2018-02-17

**Authors:** Raúl Arias-Carrasco, Yessenia Vásquez-Morán, Helder I. Nakaya, Vinicius Maracaja-Coutinho

**Affiliations:** 1 Centro de Genómica y Bioinformática, Facultad de Ciencias, Universidad Mayor, 8580745 Santiago, Chile; 2 Programa de Doctorado en Genómica Integrativa, Vicerrectoría de Investigación, Universidad Mayor, 8580745 Santiago, Chile; 3 Faculdade de Ciências Farmacêuticas, Universidade de São Paulo, São Paulo, 05508-900 Brazil; 4 Instituto Vandique, João Pessoa, 58000-000 Brazil; 5 Beagle Bioinformatics, 8320000 Santiago, Chile; 6 Advanced Center for Chronic Diseases (ACCDiS), Facultad de Ciencias Químicas y Farmacéuticas, Universidad de Chile, 8380492 Santiago, Chile

**Keywords:** RNA family, RNA structure, Noncoding RNAs, Covariance models, Web server, Tool, Pipeline

## Abstract

**Background:**

The function of many noncoding RNAs (ncRNAs) depend upon their secondary structures. Over the last decades, several methodologies have been developed to predict such structures or to use them to functionally annotate RNAs into RNA families. However, to fully perform this analysis, researchers should utilize multiple tools, which require the constant parsing and processing of several intermediate files. This makes the large-scale prediction and annotation of RNAs a daunting task even to researchers with good computational or bioinformatics skills.

**Results:**

We present an automated pipeline named StructRNAfinder that predicts and annotates RNA families in transcript or genome sequences. This single tool not only displays the sequence/structural consensus alignments for each RNA family, according to Rfam database but also provides a taxonomic overview for each assigned functional RNA. Moreover, we implemented a user-friendly web service that allows researchers to upload their own nucleotide sequences in order to perform the whole analysis. Finally, we provided a stand-alone version of StructRNAfinder to be used in large-scale projects. The tool was developed under GNU General Public License (GPLv3) and is freely available at http://structrnafinder.integrativebioinformatics.me.

**Conclusions:**

The main advantage of StructRNAfinder relies on the large-scale processing and integrating the data obtained by each tool and database employed along the workflow, of which several files are generated and displayed in user-friendly reports, useful for downstream analyses and data exploration.

**Electronic supplementary material:**

The online version of this article (10.1186/s12859-018-2052-2) contains supplementary material, which is available to authorized users.

## Background

Noncoding RNAs (ncRNAs) are present in all domains of life, playing a critical role in the fine-tuning regulation of biological processes [1]. Their mode of action varies according to the RNA family it belongs. In 2005, the Rfam database created a limited type ontology to better represent the thousand of families identified so far and stored in the database [2]. Briefly, non-coding RNA genes (Gene) are composed by bona-fide RNAs with a recognised function (e.g. CRISPR, miRNAs, ribozymes, rRNAs, snoRNAs); structured *cis*-regulatory elements (Cis-reg), are represented by structural regulatory motifs available in RNA sequences (e.g. frameshift elements, riboswitches, thermoregulators); and Intron, composed by self-splicing RNAs. The prediction of RNA families in genome or transcriptome sequences often depends on its primary sequence conservation or secondary structural motifs. Thus, several bioinformatics workflows that use third-party software were created to predict or annotate different RNA classes using sequence or structure comparisons [2–5].

Secondary structure-based methods are critical for the annotation of specific regulatory RNAs [6, 7]. These approaches employ nucleotide sequences folding and minimum free energy calculation, in order to predict most of well-known RNA families [8]. For instance, microRNAs (miRNAs) have a characteristic structure of highly paired nucleotides, forming a double strand RNA structure from a single molecule of approximately 100 nt. Further, snoRNAs (small nucleolar RNAs) present a big loop associated to their binding with ribosomal RNAs (rRNAs). These structural features are of key importance for their ab-initio prediction and classification [9–11], sometimes coupled with other features, such as nucleotides conservation and covariation [12, 13]. Tools such as Infernal [14] utilize nucleotide sequences and/or secondary structure covariance models from known RNA families, like those available in Rfam and other databases [15, 16]. These tools directly compare known RNA families and nucleotide sequences, resulting in the identification of potential novel regulatory RNAs in genome or transcriptome sequences. However, large-scale prediction and annotation of regulatory noncoding RNAs can represent a daunting task, as multiple tools are required along the process. In addition, the constant parsing and processing of intermediate files needed to run these tools impose a great obstacle for researchers with lesser computational or bioinformatics backgrounds.

To avoid these technical bottlenecks, we developed an automated pipeline named StructRNAfinder. The tool was implemented using Perl scripts and third-party software and is focused on the identification and complete annotation of regulatory RNA families from transcriptome or genome sequences. We also implemented a user-friendly web server that allows users with no bioinformatics skills to perform all analyses. Both stand-alone and web server StructRNAfinder versions can be accessed at http://structrnafinder.integrativebioinformatics.me.

## Implementation

### StructRNAfinder automated pipeline

The stand-alone tool was developed to run in Linux and requires different third-party software (i.e. Infernal [14], RNAfold [3], Rfam database [15], Krona [17], different Perl libraries), automatically downloaded and installed together with the software. All the StructRNAfinder codes were developed using Perl scripts (Additional file 1), which creates HTML pages integrating JavaScript to generate dynamic tables and graphics. The usage is simple, only requiring the definition of two input files for the comparisons. The first is the database of covariance models, used as a reference for RNA families to be found in the second input file, which is the nucleotide sequences in FASTA format. StructRNAfinder was created under GNU General Public License (GPLv3).

### Web server implementation

webStructRNAfinder server interface was implemented using PHP, HTML and Perl in an Apache2 server. We applied a FIFO pile (First In First Out) for the management of users jobs, to organise and manage the queue of submitted queries. When a user launches a job, PHP automatically creates a user-specific folder to save its results and retain the job process on the pile. When the process daemon detects a new job, it executes structRNAfinder with the FASTA sequence(s) and parameters provided by the user, updating automatically the PHP files inside the user folder. When the whole process is finished, the provided URL will be made available to the final user for 48 h. All StructRNAfinder generated HTML and output files are made available compressed in a zip file in the *Files* section.

## Results

### An automated pipeline named StructRNAfinder

To identify potential noncoding RNAs in genome or transcriptome sequences, research groups have to manually run several programs, which generate different formats of intermediate files. StructRNAfinder automates this laborious workflow, processing the data obtained by each employed tool, thus allowing non-bioinformaticians to identify and compare ncRNAs through the primary sequence and secondary structure inferences. All the files generated along the workflow are displayed in user-friendly reports and subsequently made available for downstream analyses. StructRNAfinder utilizes Infernal [14] to annotate genome/transcriptome-derived sequences to the corresponding RNA families. For data derived from next generation sequencing (NGS) studies, it is necessary to have the final sets of assembled sequences. Thus, all sequences are compared against covariance models, which represent the sequence/structural consensus alignments for each RNA family, reported to date in Rfam database [15].

**Fig. 1:**
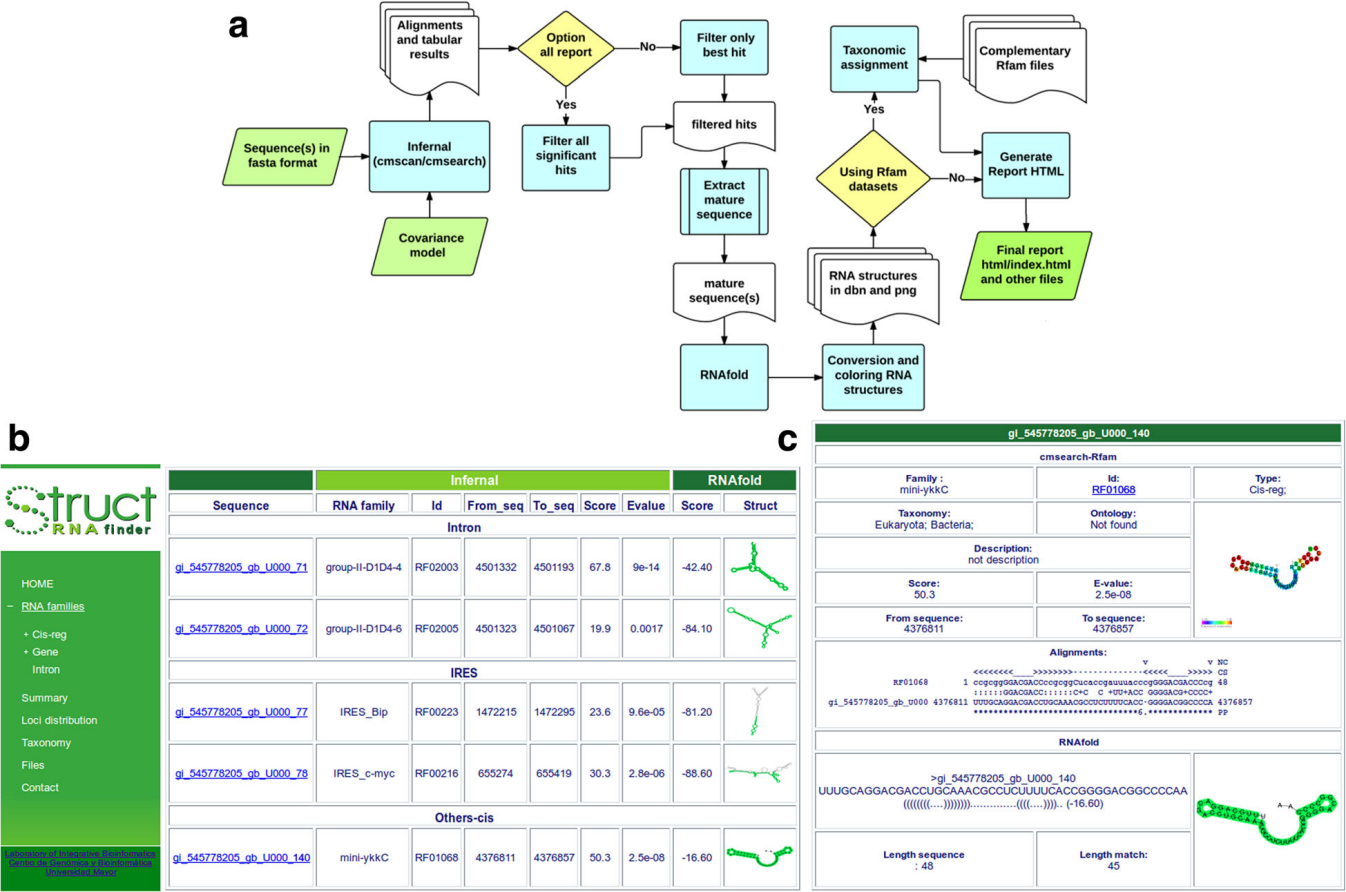
**a** Whole pipeline implemented in StructRNAfinder. Input and output files are shown in green rhomboid; intermediate third-party or in-house scripts are shown in light blue squares; together with intermediate files generated along the process, shown in white shapes. Decisions taken along the process are shown in yellow diamonds. **b** General report containing the list of predicted RNA structures in *E. coli* strain K-12. **c** Detailed table for a particular predicted RNA of interest. All information related to the covariance model comparison, secondary structure inference and complete annotations are made available in this page, including a external link to Rfam database

One issue that arises when comparing sequences and covariance models is that current tools only provide results in plain text outputs, which contains the sequence-structure alignments, positional coordinates and its statistics. No information is provided related to a potential annotation of the predicted RNA families, neither images with the potential RNA secondary structure, its description in the standard dot and bracket format, nor its functional description. StructRNAfinder automatically explores and parses Infernal alignments output, by filtering and extracting significant hits for each sequence/covariance model comparison. Based on its mapping coordinates, the tool calculates the length of alignment, the size of the input sequence and the size of the target RNA family. If necessary, the hit length from input sequence is expanded, in order to obtain a mature sequence with a similar size to that of the original Rfam secondary structure, which is used as input to RNAfold for secondary structure predictions. This tool is available in Vienna package [3], which is a widely-used suite of tools to analyse RNA structures. In the final structure, the region assigned to the alignment is highlighted in green. This procedure assures the length needed to estimate the optimal minimum energy, which is sequence- and length-dependent [18]. This secondary structure is a visual representation of the predicted structure, to be compared with those originally generated by Rfam, which is also available on the final report. The text representation of the structure generated by RNAfold and from the CM alignment are also reported. Once an RNA family is assigned, StructRNAfinder automatically retrieves all annotation information available in Rfam database for that particular RNA (i.e. family description, gene ontology, taxonomy, family secondary structure image). The general procedure performed by StructRNAfinder is explained in Fig. 1a.

### Comprehensive reports

**Fig. 2:**
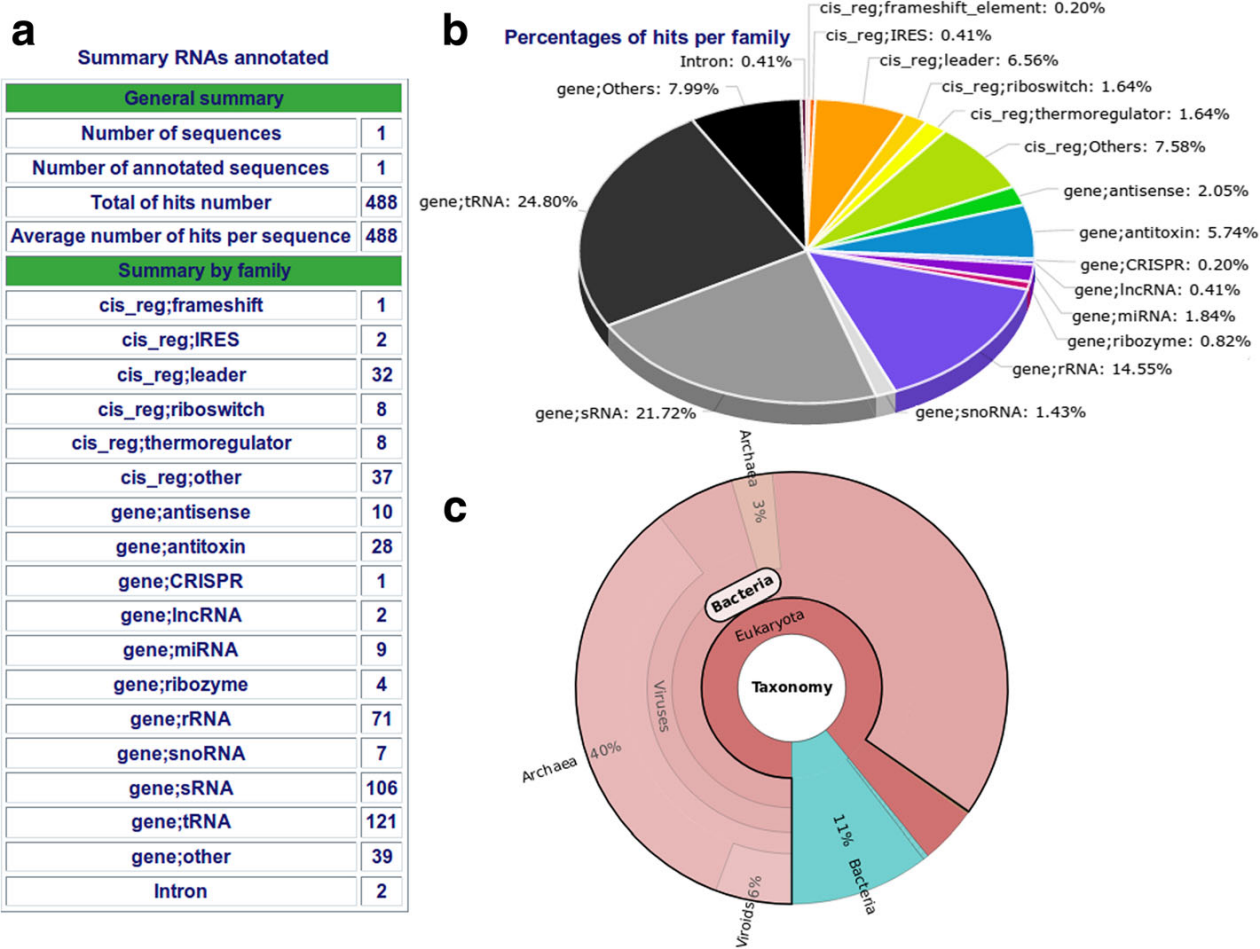
**a, b** Exemplary results of StructRNAfinder summary section. This section provides a general statistics of all identified RNA families in *E. coli* strain K-12 genome sequence. **a** Table showing the total numbers of each predicted RNA family according to Rfam nomenclature. **b** A pie-chart of the numbers shown in A. **c** A dynamic pie-chart with the taxonomic assignation of the 488 identified RNAs

The reports generated by StructRNAfinder contain the annotation and statistics for all RNA families, secondary structures, alignments, functions and taxonomic assignations identified in the input sequence(s). These reports are provided in HTML format and contain tables and figures that can be used for further data exploration. For instance, the index.html file (available in the main folder of the stand-alone version) displays a table containing all significant hits obtained from the alignment between covariance models and input sequences (Fig. 1b). The menu on the left of the table is generated dynamically according to results and allows quick navigation through the different RNA families identified in the input sequences. If users click on the hyperlink associated with each identifier hit name, a new page is opened containing the complete information of the predicted RNA (Fig. 1c), such as the full description of RNA function (if available), associated gene ontology, covariance model alignment, secondary structure predicted by RNAfold [3] and reported secondary structure from the reference RNA available in Rfam. Briefly, this page contains information extracted from Infernal, RNAfold, Rfam database and generated by our in-house Perl scripts. A general overview of statistics and a graphic representation of predicted RNA families (Fig. 2a, b) are accessible in the *Summary* section.

### Visual distribution of predicted RNA families

Users can visualize the localization of each predicted RNA along the nucleotide query sequence. This can be useful to identify potential RNA clusters generated from a unique precursor RNA or to obtain a genome-wide visualization of predicted RNAs in a whole or partial genome sequence. The *Loci distribution* section provides a visual representation of all RNA families identified along the analysed nucleotide sequence. If more than one sequence is used as input, this page will provide one image for each analysed sequence with the general distribution of RNA families.

### Taxonomic distribution and visualization

StructRNAfinder recovers the taxonomy of each predicted RNA family, according to Rfam annotation for each RNA family. We used Krona [17] to generate interactive graphics that show the abundance of all RNAs belonging to different taxonomic groups based on Rfam species annotations. In Rfam database, each RNA family is the result of multiple sequences alignments from different species. StructRNAfinder summarizes and plots the presence of all predicted RNAs according to three domains of life, plus viruses (Fig. 2c). For instance, the graphic on Fig. 2c shows the taxonomic distribution of 488 RNA families predicted on the *E. coli* strain K-12 substr. MG1655 genome sequence (accession number: U00096). This is a dynamic graphic, allowing the navigation within the number of predicted RNA available in each evolutionary branches. In this example, 53 RNA families (11% of the total) are present exclusively on the Bacteria domain (light blue in Fig. 2c); while 413 RNA families (85% of the total) are shared between Bacteria and other evolutionary branches (light red in Fig. 2c).

### Output files for downstream analysis

StructRNAfinder generates several files in different formats. They can be useful to advance downstream analyses or can supplement information available from HTML reports, obtained after running the tool. All files can be accessed on the *Files* section. Standard outputs from Infernal and RNAfold tools are available, together with other files generated by StructRNAfinder. Output files include: (i) a BED format file containing the positional coordinates of predicted structures according to the nucleotide sequences used as input; (ii) a FASTA format file containing the nucleotide sequences of the predicted RNAs; (iii) an annotation tabular file comprised of extensive information generated by StructRNAfinder. This annotation file contains the RNA family name, the RNA class, Rfam database identifiers, scores and e-values from each prediction according to Infernal and folding energies according to RNAfold, the start and end positions of each prediction on the query sequence, and finally, a functional description of the predicted RNA.

### webStructRNAfinder: An user friendly web server

**Fig. 3:**
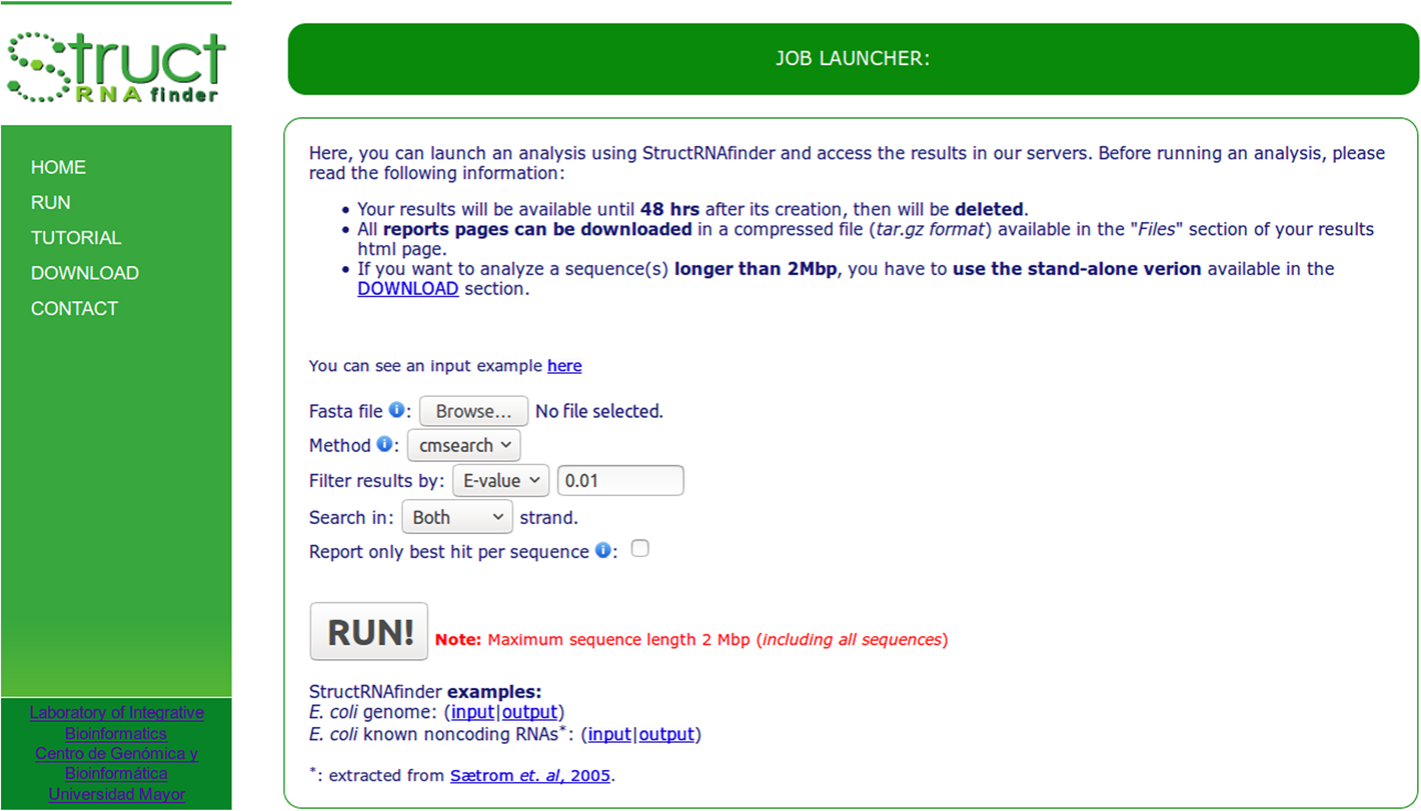
Job launcher screen showing the different parameters that a user can use when running StructRNAfinder web server

webStructRNAfinder server provides a job launcher interface (*RUN* section) where users can analyse sequences using different search methods according to their own criteria. Users are only required to provide a FASTA sequence(s) file, and to fill a small set of required parameters (Fig. 3). The parameters to choose are: (i) the Infernal search method (cmsearch, who searches the covariance models in a database composed by the input sequences; or cmscan, who searches the input sequences in a database composed by the covariance models. This difference influences the e-value calculation, due to this value mainly depend on the database size.); (ii) the cutoff filter to be used according to Infernal, based on: e-value, score, or one of the three covariance model-specific reporting thresholds (gathering, noise or trusted); (iii) the option to receive a report considering all significant hits according to selected e-value/score/CM-threshold or only the best one per sequence based on the lowest e-value; (iv) performs the search in both strands or only in one. As soon as StructRNAfinder finishes the whole analysis, the results will remain available on the provided hyperlink for 48 h. On the *Files* section, users can download a compressed file in zip format containing all output files and HTML web pages generated by the tool.

StructRNAfinder exemplary reports are made available in the *RUN* section. One with RNA families predicted in the genome of the eukaryotic human pathogen *Leishmania braziliensis* (Additional file 2); another in *E. coli* str k-12 genome (Fig. 2), both using the cmsearch Infernal method and filtered by an e-value of 0.01; and with RNA families predicted in a dataset of experimentally verified ncRNA sequences extracted from Sætrom and collaborators [19]. This last analysis predicted correctly 151 out of 154 (98.05% of the total) experimentally validated RNAs (Additional file 3).

## Discussion

In this work, we described a new automated pipeline, named StructRNAfinder, which was developed to facilitate the identification and complete annotation of regulatory RNA families available in nucleotide sequences (DNA or RNA). When predicting/annotating RNAs in nucleotides sequences, the user needs to manipulate several plain text results generated by different tools used on the process, which are difficult to visualize and manipulate in typical text viewers rather than Linux command line. The advantage of StructRNAfinder relies on processing and integrating the data obtained by each tool and database employed along the workflow, of which several files are generated and displayed in user-friendly reports, useful for downstream analyses and data exploration.

We have successfully applied the stand-alone version of this automated pipeline on analyses of the noncoding RNA content in genomic sequences from the most diverse set of organisms, covering all three domains of life, plus viruses (unpublished and published data). For instance, on the development of LeishDB [20], a reference database for *Leishmania braziliensis* genomic information; we used this tool, together with other strategies, to obtain the most comprehensive characterization of noncoding RNAs for *Leishmania* species. Analyses using genomic and transcriptomic *E. coli* datasets allowed the prediction of 488 different RNAs (cmsearch e-value cutoff of 0.01), part of 184 different Rfam families, on the genomic sequence of *E. coli* str k-12; and the correct prediction of 98.05% of experimentally validated RNA transcripts (i.e. predicted with the same functional annotation as provided by the authors [19]). The remaining 2% are related to validated RNAs in which their covariance models are not yet made available in Rfam database.

StructRNAfinder is presented in both stand-alone and web server versions, facilitating the usage for all kind of users. The web-based version allows the user-friendly prediction of RNA families available in sequences of up to 10,000,000 nucleotides (10 Mb), which is enough to predict the repertoire of regulatory RNAs in the sequenced genomes of all Archaea and most of Bacteria available in NCBI. Indeed, Rfam allows a batch sequence search on their web server. However, it does not allow the usage of different filtering options available in StructRNAfinder (i.e. Infernal search method, cutoff filters, strand-specific search, best hit per sequence selection), and its search is limited to 200,000 nucleotides (200 Kb). A table comparing webStructRNAfinder, structRNAfinder and Rfam batch search is available in Additional file 4.

The advantages of StrucRNAfinder stand-alone compared to its web-based version are the possibility to analyse large genomes, e.g. eukaryotic organisms; to perform large-scale analyses using several genomes; and to use as input the covariance models generated by the user itself, instead of those generated by Rfam. Indeed, recently, Eggenhofer and collaborators developed an unsupervised tool that allows the generation of covariance models using a single sequence as input [21]. It collects potential RNA family members from a model generated based on multiple interactions of homology searches against nucleotide sequences of taxonomy related organisms available in NCBI, and structural alignments analyses, in order to generate the most suitable covariance model for that particular RNA sequence. For instance, this is useful for structural conservation analysis of new ncRNAs found in transcriptomes, in which users can easily generate a covariance model of one or a set of transcripts of interest and search for its presence in different sets of genome sequences using the stand-alone version of StructRNAfinder.

## Conclusions

StructRNAfinder facilitates de prediction and complete functional annotation of RNA families in nucleotides sequences, by integrating different tools and databases commonly used on the prediction and functional annotation of regulatory RNAs. One main advantage over other existing tools relies on the large-scale processing and integration of the data obtained by each tool and database employed along the workflow, with useful files generated and displayed in user-friendly reports helpful for downstream analyses. The automatic generations of these reports avoid the time-consuming process of writing scripts for parsing the output and input files for the tools and databases employed, especially for users without any programming or bioinformatics skills. These features facilitate the genome-wide predictions of the complete repertoire of RNA families available in small and large genomes and also in assembled transcriptomes derived from NGS studies.

## Availability and requirements

**Project name:** StructRNAfinder.

**Project home page:** http://structrnafinder.integrativebioinformatics.me

**Operating system(s):** LINUX.

**Programming language:** Perl, HTML5, PHP5 and JavaScript.

**Other requirements:** Linux Perl package libgd-perl version 2.53, Perl package Bio::Graphics 2.4, Infernal tool version 1.1, Vienna package version 2.1.8, Rfam covariance models version 12.

**License:** GNU GPL version 3.

## Additional files

Additional file 1: Table in XLS format summarizing each developed Perl script part of StructRNAfinder tool. (XLS 18 kb)

Additional file 2: Exemplary results (figure in PNG format) of StructRNAfinder in *Leishmania braziliensis* genome. (**A)** A pie-chart of the total numbers of each predicted RNA family according to Rfam nomenclature. (**B**) Table showing the numbers shown in A. (**C**) A dynamic pie-chart with the taxonomic assignation of identified RNAs. (PNG 116 kb)

Additional file 3: (**A**) Exemplary results (figure in PNG format) of StructRNAfinder in *E. coli* validated transcripts from Sætromet al., 2005. (**A)** A pie-chart of the total numbers of each predicted RNA family according to Rfam nomenclature. (**B**) Table showing the numbers shown in A. (**C**) A dynamic pie-chart with the taxonomic assignation of identified RNAs. (PNG 120 kb)

Additional file 4: Table in XLS format comparing the features of StructRNAfinder, webStructRNAfinder and Rfam batch search. (XLS 21 kb)

## Abbreviations

BED: Browser extensible data
Cis-reg: Cis-regulatory
CM: Covariance model
CRISPR: Clustered regularly interspaced short palindromic repeats
DNA: Deoxyribonucleic acid
*E. coli*: *Escherichia coli*
FIFO: First in first out
GNU GPL: GNU general public license
GNU: GNU is not Unix
HTML: Hypertext markup language
Kb: Kilobases
*L. braziliensis*: *Leishmania braziliensis*
Mb: Megabases
miRNA: MicroRNA
NCBI: National center for biotechnology information
ncRNA: Noncoding RNA
NGS: Next generation sequencing
PHP: Hypertext preprocessor
RNA: Ribonucleic acid
rRNA: Ribosomal RNA
snoRNA: Small nucleolar RNA
URL: Uniform resource locator
