# Supplementary figures and images for: StructRNAfinder: an automated pipeline and web server for RNA families prediction
Source: BMC Bioinformatics. 2018 Feb 17;19:55. doi: 10.1186/s12859-018-2052-2 (PMC5816368; doi:10.1186/s12859-018-2052-2)

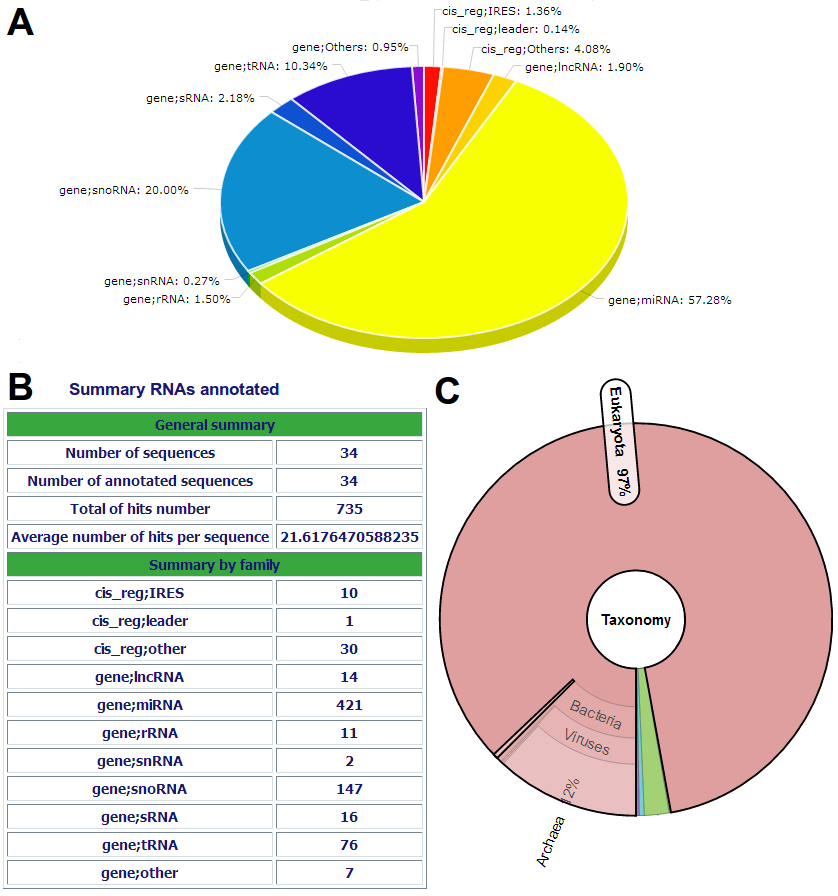

Supplement: Supplementary file 2 — Exemplary results (figure in PNG format) of StructRNAfinder in Leishmania braziliensis genome. (A) A pie-chart of the total numbers of each predicted RNA family according to Rfam nomenclature. (B) Table showing the numbers shown in A. (C) A dynamic pie-chart with the taxonomic assignation of identified RNAs. (PNG 116 kb) [file 12859_2018_2052_MOESM2_ESM.png]

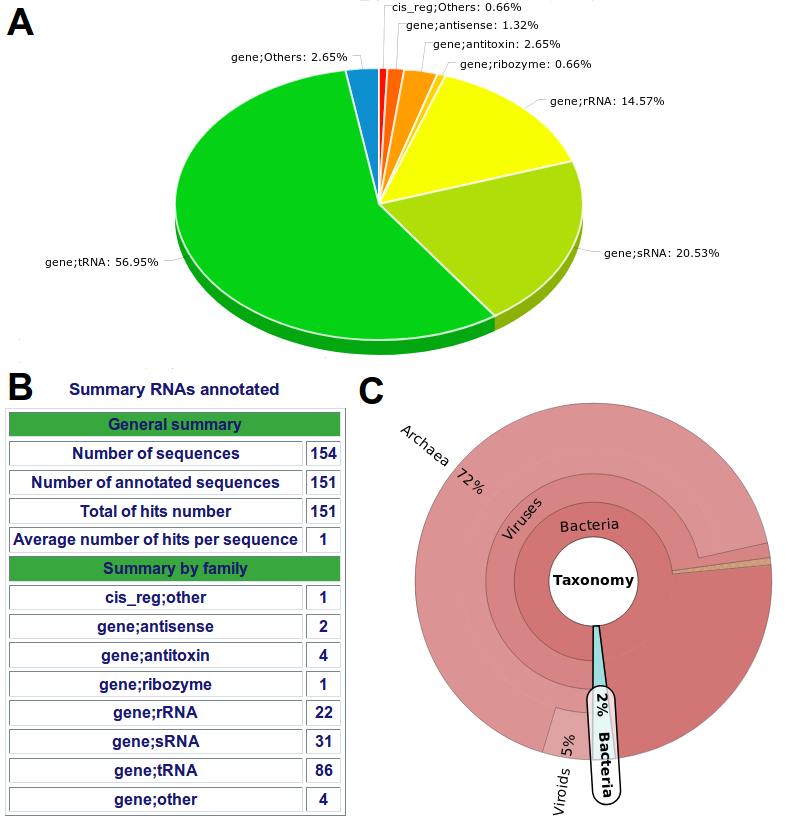

Supplement: Supplementary file 3 — (A) Exemplary results (figure in PNG format) of StructRNAfinder in E. coli validated transcripts from Sætromet al., 2005. (A) A pie-chart of the total numbers of each predicted RNA family according to Rfam nomenclature. (B) Table showing the numbers shown in A. (C) A dynamic pie-chart with the taxonomic assignation of identified RNAs. (PNG 120 kb) [file 12859_2018_2052_MOESM3_ESM.png]
